# The influence of resource-gaining capacity on mate preferences: an eye tracking study

**DOI:** 10.1186/s40359-023-01487-7

**Published:** 2023-12-18

**Authors:** Ziyue Zhao, Wei Su, Juan Hou

**Affiliations:** Department of Psychology, School of Philosophy, Anhui University, Hefei, Anhui P.R. China

**Keywords:** Resource-gaining capacity, Mate preferences, Sex differences, Eye tracking technique

## Abstract

To investigate whether resource-gaining capacity influences mate preferences, explicit (self-report data) and implicit tasks (eye tracking data) were used to explore whether individuals’ resource-gaining capacity influences mate preferences and whether there are sex differences in mate preferences under two different conditions (short-term and long-term strategies). A total of 59 college students completed a questionnaire collecting basic demographic information, the Resource-Gaining Capacity Scale and the two above tasks. The results showed that (1) in the short-term mating, individuals with higher resource-gaining capacity paid more attention to “good parent” than those with lower resource-gaining capacity, while individuals with lower resource-gaining capacity preferred “good provider” than those with higher resource-gaining capacity. (2) In the long-term mating, women valued “good provider” traits more than men, and they paid more attention to “good parent” traits than men in the short-term. In addition, no matter in the short-term or the long-term mating, men placed more value on “good genes” traits than women. (3) Compared with long-term mating, individuals of both sexes had preferences based on “good genes” in short-term mating, while they had preferences based on “good parent” and “good provider” in long-term mating compared with short-term mating. (4) Regarding explicit mate selection, “good parent” traits were most preferred by the participants, while the implicit eye tracking data indicated that participants preferred partners who were “good providers” and had “good genes”.

**Supplementary Information:**

The online version contains supplementary material available at 10.1186/s40359-023-01487-7.

## Background

Mate selection is a prerequisite for marriage and family and plays an important role in human survival and reproduction [1]. In addition, mate selection is an important decision-making process that requires individuals to process complex information and select among multiple candidates according to the multiple attributes of candidates under uncertain conditions; therefore, people show different preferences for mates. Lu, Zhu and Chang (2015) classified mate preferences according to the “3G” traits: good genes, good providers, and good parents [2]. “Good genes” refer to the individual’s biological characteristics, mainly including appearance and body shape. Good genes help the individual adapt to the natural environment and are passed down through evolution, and they are closely related to humans’ survival ability and instincts. “Good provider” traits reflect the individual’s social and economic characteristics, such as the individual’s economic ability, power and social class, while “Good parent” traits mainly refer to the individual’s personality characteristics, including kindness, honesty, love for children and so on, and are mainly related to parental investment.

There are differences in mate preferences among individuals, and the factors associated with these differences generally include sex, resource conditions and personality characteristics [3, 4]. Among these factors, the individual’s resource conditions play an important role [5]. In general, as an individual’s resources increase, so does the strictness of his or her criteria for choosing a mate [6, 7]. Buss and Shackelford (2008) demonstrated that women who perceive that they have high mate value are more selective in terms of partner choice; similar findings were [8]observed in men [9]. South (1991) found that as one’s salary and educational level increased, one’s mate-selection criteria became stricter [7]. Yong and Li (2012) found that individuals with more resources preferred looks in mate choice [10]. However, previous studies of individual resource conditions have often considered only the financial wealth that the individual already possesses, while an individual’s resource condition incudes not only the resources that the individual possesses but also his or her economic potential in the future, that is, his or her resource-gaining capacity. This is especially true for young people, who currently have limited resources, and thus, the differences between them are small. Therefore, the most effective index to investigate the role of resource conditions in mating trade-offs is resource-gaining capacity [5]. As an important factor affecting an individual’s survival and development, resource-gaining capacity also affects an individual’s mate-selection behavior. A number of studies have shown that men’s resources are an important factor for women in choosing a mate [11, 12]; women prefer men with superior resource conditions, and women with lower resource-gaining capacity tend to value men’s resource conditions more when choosing a mate. Other studies have shown that men with higher resource-gaining capacity increase their demands for women’s resources when there are more women than men [5]. Therefore, this study explores the effect of individual resource-gaining capacity on mate preferences.

In addition, there are sex differences in the effects of resources on mate preferences. According to the structural powerlessness hypothesis, men in most cultures control social power and resources, while women have limited resources; therefore, women have a greater preference for men who possess more social resources [3, 13]. In addition, mating gradient theory refers to the influence of the larger social structure on the family structure, with men expected to have high status than their female partners. According to this theory, during socialization, people develop different sex role expectations for their partners [14]. As a result, when women have a greater ability to gain resources, their preferences for male resources will be reduced. When men and women have equal rights and resources, women will demand less of their partner’s economic condition, and sex differences in mate preferences will be reduced [6]. Studies have shown that women value their partners’ socioeconomic status more than men when they have fewer resources and that women with more financial resources place less emphasis on their partner’s socioeconomic status than those with fewer resources [15, 16]. However, some studies have reached different conclusions; for example, a study of married couples in the United States found that high-earning women preferred higher-earning men than women with average income [11]. Other research has shown that women with high socioeconomic status value “good fathers” more than “good providers” or “good genes” [2]. For men, a study found that men do not value the economic resources of women when choosing a mate, regardless of their socioeconomic status [17]. According to evolutionary psychology, the ultimate goal of mating is the survival of one’s genes; as a result, men are more likely to choose a partner based on visual cues such as the woman’s looks and body shape that are relevant to her fertility [18]. By using the mate budget paradigm, a study showed that male preferences were based on women’s physical attractiveness while female preferences were based on men’s socioeconomic status under low resource conditions, suggesting that when choosing a mate, these two traits are important [19]. However, a study by Deng (2015), a Chinese researcher, found that for both Chinese female and male graduates, the necessities were partner personality and feeling of love, while physical attractiveness and economic conditions were luxuries [20]. There are still some different conclusions about the effect of resource conditions on both sexes.

Why have previous studies not come to a unanimous conclusion? We believe that one reason is the research background and cultural differences. Most of the studies mentioned above were conducted in Western cultural contexts and may have been affected by unique social and cultural factors. For example, a study found that when choosing a mate, Chinese people placed more importance on status, family background and income, while Americans placed more emphasis on agreeableness and attractiveness [21]. The second reason may be that most previous studies did not compare long-term mating (for marriage and reproduction) and short-term mating (i.e., casual sexual activity) in a study. For example, some studies found that women under economic pressure generally pay more attention to resource clues when choosing a partner [19, 22, 23], while other studies pointed that women prioritize cues such as man’s personality, health and conscientiousness when choosing a mate [24, 25]. There have inconsistent results because long-term and short-term mate selection were not compared in the same study. According to sexual strategies theory, individuals seeking a mate attempt to maximize the personal resources derived from the relationship. And due to different “adaptive problems” during the course of evolution, men and women develop different sexual strategies, including short-term and long-term strategies [26]. The long-term strategy is based on building lasting, long-term relationships, while the short-term strategy emphasizes attracting a mate who provides “good genes” or health [27]. Although some studies have compared the two, they are both focused on the field of attractiveness [28, 29]. Therefore, it is necessary to explore the impact of resource-gaining capacity on mate preferences in the condition of long-term mating and short-term mating. Additionally, due to the relaxation of traditional views on sexual relationships and negative attitudes concerning whether college romances will result in marriage, short-term mating has become more common; therefore, it is need to examine short-term and long-term mating in-depth in the context of Chinese culture [30, 31].

In general, individuals are influenced by sociocultural and evolutionary forces, both explicit and implicit, when choosing a mate. Self-reports are the most widely used method to examine the influence of the social culture on mate selection. This method measures participants’ conscious preferences, and participants tend to think carefully about their answers, which may be more socially acceptable and less likely to fully reflect their true feelings. Studies have shown that social and cultural forces can mask people’s true mate preferences and that indirect methods can better detect people’s implicit and true mate preferences [4]. Therefore, researchers have developed a series of indirect experimental methods to measure individuals’ truer mate preference, such as the “Go/No Go” association test, information board technology and priming methods. These indirect methods can reflect the strength of automatic associations between an individual’s attitude and the presented words and can better reflect the influence of biologically evolved instincts on mate selection. Although the implicit association test (IAT) and the “Go/No Go” association test can avoid the influence of sociocultural factors to some extent, they are still being questioned; for example, the IAT may be influenced by the sequence of the two associated tasks of the IAT and may be susceptible to the individual’s current state [32, 33]. However, the eye tracking technique is more advantageous in this aspect; the participant only needs to look at the visual stimulation autonomously in his or her natural state, avoiding social appraisal. In addition, it is difficult for participant to control some of their eye movements. Therefore, the eye tracking index can reflect participants’ preferences for stimulus materials to a great extent. Accordingly, this study intends to use eye tracking technology to explore individual implicit mate preferences and to understand the potential psychological process of individual mate selection. In previous studies of mate selection, eye tracking techniques have been used to investigate the effects of biological features such as facial attractiveness and facial similarity on mate selection [34–36]. For example, eye tracking measures have been used to assess women’s preferences for male facial masculinity [37, 38] and the relationship among gaze behavior, perceived physical attractiveness and the shoulder to hip ratio (SHR) of individuals [39]. Eye-tracking tasks can also provide evidence of the mechanisms underlying formidability assessment [40]. In addition, He and Hu (2011) explored the processing mechanism of mate choice with eye tracking technology [41]. In this study, the eye tracking technique was used to predict the intrinsic cognitive process of individuals by recording fixation counts, fixation dwelling time and mean pupil size. To examine the effects of both sociocultural and evolutionary forces on individual mate selection, implicit eye tracking experiments were combined with explicit self-report methods. The purpose of this study was to explore the differences between implicit mate preference and explicit mate preference to draw a more comprehensive and accurate conclusion.

In summary, based on the “3G” traits, the structural powerlessness hypothesis, mating gradient theory and sexual strategies theory, this study aimed to explore whether individuals’ resource-gaining capacity influences mate preferences and whether there are sex differences in mate preferences under two different mating conditions, i.e., short-term and long-term mating strategies. Based on the different purposes of long-term and short-term mating, we hypothesize the following: (1) Resource-gaining capacity will affect individual’s mate preferences. Individuals with higher resource-gaining capacity will pay more attention to “good genes” and “good parent” than those with lower resource-gaining capacity; individuals with lower resource-gaining capacity, on the other hand, will place more value on “good provider” than those with higher resource-gaining capacity. (2) There will be sex differences in the effects of mating strategy on mate preferences. In both the short-term and long-term mate selection, men pay more attention to “good genes” traits than women. However, in long-term mate selection, women pay more attention to “good provider” traits than men, and in short-term mate selection, they pay more attention to “good parent” traits than men. (3) There will be distinctions between mating strategy on individual’s mate preferences. Compared with long-term mating, individuals of both sexes will have preferences based on “good genes” in short-term mating, while they will have preferences based on “good parent” and “good provider” in long-term mating compared with short-term mating. (4) There will be some differences between implicit and explicit test results. In explicit mate selection, influenced by traditional Chinese culture and values such as “paying more attention to conduct than money”, individuals will prefer “good parent” traits; however, in implicit mate selection, influenced by evolutionary instincts, men will prefer “good genes” traits, and women will prefer “good provider” traits.

## Methods

### Participants

G*Power 3.1.9.2 was used to estimate the planned sample size (α = 0.05, 1–β = 0.95). According to the standard for a medium effect size (effect size f = 0.25), the total number of participants required was 40 [42]. Considering a sample loss rate of approximately 10%, 70 participants (34 males and 36 females) were recruited. The age distribution of participants ranged from 18 to 25 years old (*M* = 20.00, *SD* = 2.12). All participants had normal or corrected-to-normal vision and no eye diseases. Before the formal experiment, participants were asked to complete a scale developed by Wang, Yao and Zhou (2015) to assess their sexual orientation [43]. Participants with scores greater than or equal to 5 were excluded. According to the results of the test, 5 participants with same-sex attraction were excluded, and the 65 participants who took part in the formal experiment all exhibited opposite-sex attraction. In the available data, the average number of past romantic relationships was 2 for male participants (*SD* = 1.184) and 2 for female participants (*SD* = 1.491). However, some eye tracking data were lost due to technical difficulties and other factors; thus, 59 participants (28 males and 31 females) were included in the final analysis.

G*Power 3.1.9.2 was used for post-hoc calculations of statistical power (total sample size = 59, α = 0.05). According to the standard for a medium effect size (effect size f = 0.25), the power (1–β) was 0.997.

### Ethics statement

This study was approved by the Human Research Ethics Committee of Anhui University, China, and followed the principles expressed in the Declaration of Helsinki. Each participant signed an informed consent form after receiving an explanation of the study’s purpose and procedure. All the participants were older than 18 years of age, and before the experiment, all participants completed the experimental informed consent form. Participants were given 50 RMB (approximately 7.356 USD) as an incentive to participate in the study.

### Design

The experiment used a 2 × 2 × 2 × 3 mixed design. The independent variables were resource-gaining capacity (high vs. low), sex (male vs. female), mating strategy (long-term mating vs. short-term mating) and mating dimension (good genes vs. good providers vs. good parents). The dependent variables were the results of explicit and implicit mate preferences. The explicit mate preference task involved selecting different trait words according to their importance; scores were assigned according to the order of selection (from first to last) in three different dimensions. The implicit task involved recording the individual’s response (eye movements) to different trait words, and the fixation count, average fixation duration and average pupil size of participants were recorded as indicators of implicit mate selection. The fixation count is the sum of the fixation points of each trait word (region of interest), which could effectively reflect the visual attention and value of the reading material [44, 45]. The average fixation duration refers to the average duration of all fixation points of different dimensions, which can better reflect the duration of encoding, processing and meaning extraction of current fixation information [46, 47]. Average pupil size is an index of how interested an individual is in the current area [48, 49].

### Research materials

#### Heterosexual–homosexual rating scale

We used the scale developed by Wang et al. (2015) to screen participants according to sexual orientation. Participants were asked to rate their sexual orientation on a nine-point item (from 1 to 9, indicating “No same-sex attraction at all” to “Extremely strong same-sex attraction”), with a score greater than or equal to 5 indicating same-sex attraction [43].

#### Basic demographic information questionnaire

A basic demographic information questionnaire was designed to collect the participants’ personal information, such as age, sex, and emotional state.

#### Resource-gaining capability scale

We used the two-item scale developed by Wang et al. (2017). In this scale, participants rate their future “earning power” on a scale from 1 (“very poor earning power”) to 6 (“very good earning power”); participants also rate their “career potential” on a six-point scale (1 = “very low career potential” to 6 = “very high career potential”) [5]. The higher the score was, the higher the resource-gaining capability. The Cronbach’s α coefficient was 0.86.

#### Word material for cue preferences

According to the 3G traits, that is, good genes, good parents and good providers, 30 words were selected from 45 male two-character words compiled by Tian, Zhang and Sun (2019), with 10 words in each dimension. According to the male trait words compiled by Tian and combined with the opinions of the expert group, the researcher revised the corresponding 30 female trait words, with 10 words in each dimension [50]. Thirty words representing the sexual traits of males and females were processed with Photoshop software separately according to the requirements of the experiment. The 30 words were divided into 6 groups, and each group contained 5 words from different dimensions. All the words were edited and adjusted according to the experimental procedure of measuring the preferences of men and women.

#### Eye tracker

The experiment used an Eyelink 1000 desktop eye tracker system (SR Research Ltd., Mississauga, Ontario, Canada) for eye tracking. The stimulus was displayed on a 15.6-inch display with a resolution of 1024 × 768 and a screen refresh rate of 60 Hz. The display was 55 cm from the chin rest, which reduced head movement and ensured comfort. Experiment Builder software (version 2.3.38) adapted to the Eyelink 1000 eye tracker was used to write the experimental program. All participants viewed the stimuli with both eyes, but only the position of the right eye was tracked and recorded. The eye tracker was calibrated with the nine-point calibration method. The average fixation duration, fixation counts and average pupil size were recorded.

### Procedures and measures

At the beginning of the experiment, the participants were asked to complete the basic demographic information questionnaire, the Heterosexual-Homosexual Rating Scale and the Resource-Gaining Capability Scale.

After completing the questionnaires, the participants started the mate preference experiment, which was divided into a long-term-mating experiment and a short-term-mating experiment. To obtain the results of both explicit and implicit mate selection tests, the mating preference measurement paradigm and visual search paradigm used by Tian et al. (2012; 2019) were used, the number of trait words used in the test was reduced to 5, and the pictures in the visual search task were replaced with trait words [23, 50]. The male trait words came from 30 words selected from 45 male two-character words compiled by Tian et al. (2019), with 10 words in each dimension; female trait words were selected the researcher according to the male trait words compiled by Tian and the opinions of the expert group, for a total of 10 words in each dimension [50]. Under the condition of long-term mating, the participants carried out the experiment independently after experimenter calibrated the eye tracker and explained the precautions. First, participants saw the following instructions on the screen: “Next, there will be 6 groups of words related to partner traits on the screen, and each group contains 5 words. Please browse each group of words and select the trait words you think you would look for in a long-term marriage partner in order of importance. After you understood the procedure, click the Start button to begin the experiment”. After the participants clicked the “Start” button, the first set of trait words appeared on the screen. The first word they clicked scored 5 points, the second scored 4 points, and so on. The fifth word scored 1 point. To avoid a gaze preference in the participants, the positions of the trait words from were balanced three dimensions so that the probabilities of different dimensional traits appearing in all directions were the same. Each group of 5 trait words belonged to different dimensions, and 6 groups of words were presented randomly. After the participants had selected 5 trait words, the next group of words was presented, and the experiment finished after 6 groups of words were presented.

The short-term mating procedure was consistent with the long-term mating procedure except for the following instructions: “Next, there will be 6 groups of words related to partner traits on the screen, and each group contains 5 words. Please browse each group of words and select the trait words you think you would look for in a short-term relationship rather than a marriage partner in order of importance. After you understand the procedure, click the Start button to begin the experiment”.

**Fig. 1:**
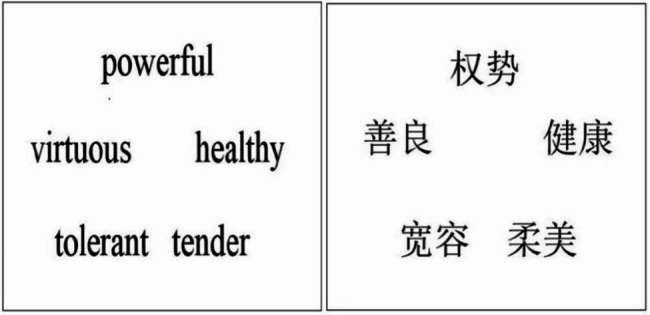
Example of trait word arrangement (in English and in Chinese)

**Fig. 2:**
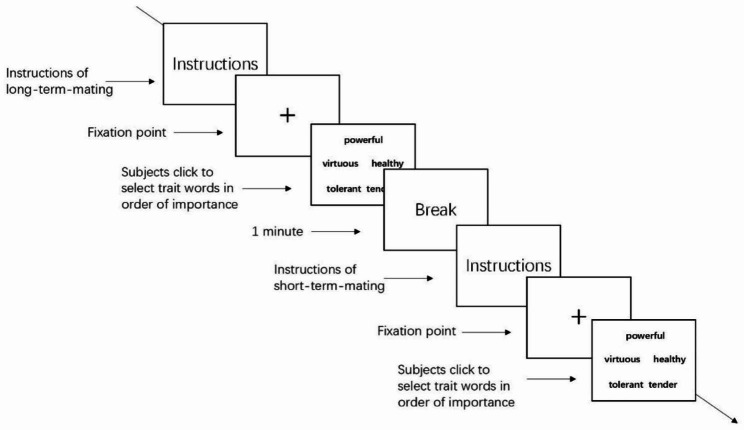
Flow chart of the mate preference experiment

To eliminate the effect of preferential fixation on the center of the screen, the distribution of the five trait words was circular (see Fig. 1), the resolution of the words was 80 × 40, the distance between words was 195 pixels, the distance between the monitor and the eyes was approximately 60 cm, and the angle of view of each stimulus was approximately 2.8° × 1.4°. Each participant completed both long-term and short-term mating decisions, with the order balanced. There was a 1-minute break between long-term-mating and short-term-mating tasks. The experimental procedure is shown in Fig. 2.

## Results

### Explicit mate preference

For the self-rating scale on resource-gaining capability, referring to the research of Wang et al. (2017), those with a score less than or equal to 8 were classified as the low-score group, and those with a score higher than 8 were classified as the high-score group [5]. With mating strategy, resource-gaining capacity, sex and mating dimension as the independent variables and participants’ preferences for different mating dimensions as the dependent variables, a mixed-design repeated-measures ANOVA was carried out, where *η*_*ρ*_^*2*^ was used to indicates the magnitude of the effect size; a value less than 0.06 is a small effect size, 0.06 ~ 0.14 is a medium effect size, and greater than 0.14 is a large effect size [51].

First, a mixed-design repeated-measures ANOVA was carried out for between-group resource-gaining capacity (high vs. low), sex (male vs. female) and within-group mating strategy (long-term mating vs. short-term mating), mating dimension (good genes vs. good providers vs. good parents). Descriptive statistics of mating dimensions are shown in Table 1. Mauchly’s test of sphericity met, so the test of within-subjects effect result was reported and results are shown in Table 2. The results showed that the main effect of the mating dimension was significant, *F* (2, 110) = 63.113, *p* < 0.001, *η*_*ρ*_^*2*^ = 0.534. The participants scored the highest in the “good parents” dimension, followed by the “good providers” dimension and the “good genes” dimension. There were no significant differences between the “good provider” and “good genes” dimensions,, while the main effects of the mating strategy (*F*(1, 55) = 0.349, *p* = 0.557, *η*_*ρ*_^*2*^ = 0.006), resource-gaining capacity (*F*(1, 55) = 0.349, *p* = 0.557, *η*_*ρ*_^*2*^ = 0.006) and sex (*F*(1, 55) = 0.349, *p* = 0.557, *η*_*ρ*_^*2*^ = 0.006) were not significant.

**Table 1:** Descriptive statistics of the scores

Mating strategy	Mating dimension	Sex	Low-score group		High-score group	
			*M*	*SD*	*M*	*SD*
Long-term mating	Good genes	Male	25.17	3.545	25.23	4.047
		Female	22.06	3.838	23.73	2.282
	Good parents	Male	38.17	4.792	38.27	3.966
		Female	35.81	3.487	36.4	4.778
	Good providers	Male	26.67	6.976	26.77	4.597
		Female	32.13	4.978	29.87	5.357
Short-term mating	Good genes	Male	31	6.229	29.45	4.501
		Female	26.38	5.005	25.47	4.549
	Good parents	Male	30.83	6.853	36.05	5.296
		Female	35.25	3.958	38.13	3.603
	Good providers	Male	28.17	9.432	24.5	4.114
		Female	28.38	4.646	26.4	3.996

**Table 2:** Analyses of the interaction of Mating strategy and Mating dimension on Sex and Resource-gaining capacity

	Mating strategy			Mating dimension			Mating strategy × Mating dimension		
	*F*	*p*	*η*_*ρ*_^*2*^	*F*	*p*	*η*_*ρ*_^*2*^	*F*	*P*	*η*_*ρ*_^*2*^
Sex	0.349	0.557	0.006	5.032	0.008	0.084	6.125	0.003	0.1
Resource-gaining capacity	0.349	0.557	0.006	2.376	0.098	0.041	2.994	0.054	0.052
Sex × Resource-gaining capacity	0.349	0.557	0.006	0.153	0.859	0.003	0.896	0.441	0.016

The interaction between mating dimension and mating strategy was significant, *F* (2, 110) = 13.953, *p* < 0.001, *η*_*ρ*_^*2*^ = 0.202. The simple effect test showed that for the “good genes” dimension, the score of the short-term mating strategy was significantly higher than that of the long-term mating strategy (*F* (1, 55) = 33.416, *p* < 0.001, *η*_*ρ*_^*2*^ = 0.378). The score of the long-term mating strategy in the “good parent” dimension (*F* (1, 55) = 7.943, *p* = 0.007, *η*_*ρ*_^*2*^ = 0.126) and the “good provider” dimension (*F* (1, 55) = 5.476, *p* = 0.023, *η*_*ρ*_^*2*^ = 0.091) was significantly higher than that of the short-term mating strategy.

The interaction between the mating strategy, mating dimension and sex was significant, *F* (2, 110) = 6.125, *p* = 0.003, *η*_*ρ*_^*2*^ = 0.1. The simple effect test showed that in the “good genes” dimension, the score of men for the long-term (*F* (1, 55) = 4.864, *p* = 0.032, *η*_*ρ*_^*2*^ = 0.081) and short-term mating strategy (*F* (1, 55) = 9.304, *p* = 0.004, *η*_*ρ*_^*2*^ = 0.145) was significantly higher than that of women. The score of women in the “good parent” dimension for the short-term mating strategy (*F* (1, 55) = 5.496, *p* = 0.023, *η*_*ρ*_^*2*^ = 0.091) and in the “good provider” dimension for the long-term mating strategy (*F* (1, 55) = 8.07, *p* = 0.006, *η*_*ρ*_^*2*^ = 0.128) was both significantly higher than that of men.

The interaction among the mating strategy, mating dimension and resource-gaining capacity was marginally significant, *F* (2, 110) = 2.994, *p* = 0.054, *η*_*ρ*_^*2*^ = 0.052. The simple effect test showed that for the short-term strategy, the score of high resource-gaining capacity group was significantly higher than that of low resource-gaining capacity group in the “good parent” dimension (*F* (1, 55) = 8.514, *p* = 0.005, *η*_*ρ*_^*2*^ = 0.134); the score of low resource-gaining capacity group was marginally significantly higher than that of high resource-gaining capacity group in the “good provider” dimension (*F* (1, 55) = 3.806, *p* = 0.056, *η*_*ρ*_^*2*^ = 0.065).

### Implicit mate preference

The fixation counts, average fixation duration and average pupil size of each dimension were calculated. With the resource-gaining capacity, sex, mating strategy and mating dimension as the independent variables and the implicit mate-selection results (average fixation duration, fixation count and average pupil size) of the participants in the three mating dimensions as the dependent variables, mixed-design repeated-measures ANOVA was carried out. Descriptive statistics of eye tracking variables are shown in Table 3. Analyses of the interaction of Mating strategy and Mating dimension on Sex and Resource-gaining capacity are shown in Table 4.

**Table 3:** Descriptive statistics of the eye tracking data

Mating strategy	Eye movement index	Mating dimension	Sex	Low-score group		High-score group	
				M	SD	M	SD
Long-term mating	Fixation count	Good parents	Male	69.00	33.472	62.45	24.14
			Female	82.69	47.549	61.53	21.534
		Good genes	Male	81.50	40.678	69.77	27.054
			Female	85.37	30.091	68.27	22.521
		Good providers	Male	90.33	41.268	68.45	31.426
			Female	89.06	44.834	65.67	21.602
	Average fixation duration	Good parents	Male	2234.79	874.874	1790.13	800.611
			Female	2306.20	1378.135	1848.62	654.971
		Good genes	Male	2712.96	1080.237	2047.72	860.705
			Female	2380.89	872.326	2175.48	810.344
		Good providers	Male	2527.89	885.47	2055.57	1001.486
			Female	2392.16	1236.728	1971.99	634.169
	Average pupil size	Good parents	Male	2475.05	846.117	2259.50	772.194
			Female	2332.06	555.32	2379.67	898.41
		Good genes	Male	2531.57	798.743	2326.13	742.872
			Female	2349.66	523.136	2418.31	859.487
		Good providers	Male	2604.36	667.304	2301.81	770.14
			Female	2337.88	525.521	2423.10	872.478
	Fixation count	Good parents	Male	53.83	13.949	59.50	26.468
Short-term mating			Female	76.38	30.72	48.67	19.5
		Good genes	Male	55.50	18.855	62.18	25.867
			Female	81.25	30.366	60.33	20.632
		Good providers	Male	59.17	20.692	64.09	27.248
			Female	77.00	30.774	54.47	15.579
	Average fixation duration	Good parents	Male	1672.09	637.911	1708.96	966.625
			Female	2013.95	1019.175	1341.98	384.844
		Good genes	Male	1731.56	640.701	1839.12	904.963
			Female	2227.27	898.238	1642.02	408.034
		Good providers	Male	1703.22	645.391	1854.41	864.858
			Female	2161.10	967.157	1591.22	339.134
	Average pupil size	Good parents	Male	2599.02	688.415	2313.71	695.656
			Female	2414.50	620.083	2431.38	871.001
		Good genes	Male	2679.35	636.479	2343.51	727.867
			Female	2422.64	653.049	2489.94	870.696
		Good providers	Male	2735.50	640.282	2353.91	721.928
			Female	2394.83	631.048	2457.56	878.522

**Table 4:** Analyses of the interaction of mating strategy and mating dimension on sex and resource-gaining capacity

Eye movement index		Mating strategy			Mating dimension			Mating strategy × Mating dimension		
		*F*	*p*	*η*_*ρ*_^*2*^	*F*	*p*	*η*_*ρ*_^*2*^	*F*	*p*	*η*_*ρ*_^*2*^
Fixation count	Sex	0.379	0.541	0.007	2.308	0.104	0.04	2.007	0.139	0.035
	Resource-gaining capacity	0.812	0.371	0.015	1.554	0.216	0.027	1.907	0.153	0.034
	Sex × Resource-gaining capacity	1.584	0.213	0.028	1.535	0.22	0.027	0.2	0.819	0.004
Average fixation duration	Sex	0.323	0.572	0.006	0.093	0.991	0.002	2.372	0.098	0.041
	Resource-gaining capacity	0.716	0.401	0.013	0.265	0.768	0.005	0.202	0.818	0.004
	Sex × Resource-gaining capacity	3.827	0.056	0.065	1.297	0.277	0.023	1.112	0.332	0.02
Average pupil size	Sex	0.094	0.76	0.002	3.974	0.025	0.128	0.714	0.494	0.026
	Resource-gaining capacity	0.429	0.515	0.008	1.877	0.163	0.065	0.11	0.896	0.004
	Sex × Resource-gaining capacity	0.194	0.661	0.004	3.06	0.055	0.102	0.794	0.457	0.029

1. Fixation count

With resource-gaining capacity, sex, mating strategy and mating dimension as the independent variables, repeated-measure ANOVA was carried out with the fixation count of different mating dimensions as the dependent variables. Mauchly’s test of sphericity met, so the test of within-subjects effect result was reported. The results showed that the main effect of the mating strategy was significant, *F*(1,55) = 7.107, *p* = 0.01, *η*_*ρ*_^*2*^ = 0.114. The number of times the participants looked at the trait words in the long-term strategy was significantly more than that in the short-term strategy. The main effect of the mating dimension was significant, *F*(2,110) = 13.929, *p* < 0.001, *η*_*ρ*_^*2*^ = 0.202. The number of times the participants looked at the trait words of the “good parent” dimension was significantly less than that of the other two dimensions, and there were no significant differences in the number of times the participants looked at the trait words between the “good genes” and “good provider” dimensions. The main effect of resource-gaining capacity was marginally significant (*F* (1, 55) = 3.662, *p* = 0.061, *η*_*ρ*_^*2*^ = 0.062), and the fixation counts in the low resource-gaining capacity group were significantly higher than those of high resource-gaining capacity group. The main effects of sex was not significant, *F* (1, 55) = 0.445, *p* = 0.503, *η*_*ρ*_^*2*^ = 0.008.

(2) Average fixation duration

With resource-gaining capacity, sex, mating strategy and mating dimension as the independent variables, repeated-measure ANOVA was carried out with the average fixation duration of different mating dimensions as the dependent variables. Mauchly’s test of sphericity met, so the test of within-subjects effect result was reported. The results showed that the main effect of mating strategy was significant, *F*(1,55) = 13.684, *p* = 0.001, *η*_*ρ*_^*2*^ = 0.199. The average fixation duration of trait words in the long-term strategy was significantly higher than that in the short-term strategy. The main effect of different mating dimensions was significant, *F* (2, 110) = 16.326, *p* < 0.001, *η*_*ρ*_^*2*^ = 0.229. The main effect of the mating dimension was significant. The average fixation duration of trait words in the “good parent” dimension was significantly less than that in the other two dimensions, and there was no significant difference in the average fixation duration of trait words between the “good genes” and “good provider” dimensions. The main effect of resource-gaining capacity (*F* (1, 55) = 2.517, *p* = 0.118, *η*_*ρ*_^*2*^ = 0.044) and sex was not significant (*F* (1, 55) = 0.04, *p* = 0.948, *η*_*ρ*_^*2*^ < 0.001).

(3) Average pupil size

With resource-gaining capacity, sex, mating strategy and mating dimension as the independent variables, repeated-measure ANOVA was carried out with the average pupil size of different mating dimensions as the dependent variables. Mauchly’s test of sphericity failed, so multivariate test results were reported. The results showed that the main effect of mating dimension was significant (*F*(2, 54) = 7.418, *p* = 0.001, *η*_*ρ*_^*2*^ = 0.216), and that the average pupil size when the participants looked at the trait words of the “good parent” dimension was significantly smaller than that for the other two dimensions; there was no significant difference in the mean pupil size between the “good genes” and “good provider” dimension. The main effects of resource-gaining capacity (*F* (1, 55) = 0.297, *p* = 0.588, *η*_*ρ*_^*2*^ = 0.005) and sex (*F* (1, 55) = 0.071, *p* = 0.791, *η*_*ρ*_^*2*^ = 0.001) were not significant.

The interaction between sex and mating dimension was significant, *F* (2, 54) = 3.974, *p* = 0.025, *η*_*ρ*_^*2*^ = 0.128. The simple effect test showed that the average pupil size of men when they looked at the “good parent” traits was significantly smaller than that when they looked the “good genes” and “good provider” traits, *F* (2, 54) = 6.877, *p* = 0.002, *η*_*ρ*_^*2*^ = 0.203. The interaction of resource-gaining capacity, sex and mating dimension was marginally significant, *F* (2, 54) = 3.06, *p* = 0.055, *η*_*ρ*_^*2*^ = 0.102. Further simple effect tests showed that in the low resource-gaining capacity group, the average pupil size of men when they were looking at the “good provider” traits was significantly larger than that at “good genes” traits and the “good parent” traits, *F* (2, 54) = 5.426, *p* = 0.007, *η*_*ρ*_^*2*^ = 0.167. In the high resource-gaining capacity group, the average pupil size of men when they were looking at the “good genes” traits was significantly larger than that at the “good parent” traits, *F* (2, 54) = 4.283, *p* = 0.019, *η*_*ρ*_^*2*^ = 0.137. In the high resource-gaining capacity group, the average pupil size of women when they were looking at the “good genes” traits was marginally larger than that at the “good parent” traits, *F* (2, 54) = 3.332, *p* = 0.043, *η*_*ρ*_^*2*^ = 0.11.

## Discussion

Based on the “3G” traits, the structural powerlessness hypothesis, mating gradient theory and sexual strategies theory, we developed an experiment including explicit (i.e., self-report data) and implicit measurement (i.e., eye tracking data). In Chinese culture, we examined whether individuals’ resource-gaining capacity affects mate preferences under long-term and short-term mating conditions, and whether there are sex differences in the impact of resource-gaining capacity on mate preferences.

The results of this study are as follows. First, in the short-term mating, individuals with higher resource-gaining capacity paid more attention to “good parent” than those with lower resource-gaining capacity, while individuals with lower resource-gaining capacity preferred “good provider” more than those with higher resource-gaining capacity. Second, in the long-term mating, women valued “good provider” traits more than men, and they paid more attention to “good parent” traits than men in the short-term. In addition, no matter in the short-term or the long-term mating, men placed more value on “good genes” traits than women. Third, compared with long-term mating, individuals of both sexes had preferences based on “good genes” in short-term mating, while they had preferences based on “good parent” and “good provider” in long-term mating compared with short-term mating. Fourth, in explicit mate selection, “good parent” traits were most strongly preferred by individuals, while the implicit eye tracking data indicated that individuals preferred mates with “good provider” and “good genes” traits.

### The difference of mate preference under different resource-gaining capacity

Under the short-term mating condition, our study found that individuals with lower resource-gaining capacity preferred partners with “good provider” traits more than those with higher resource-gaining capacity. Social exchange theory (Edward, 1969) suggested that the selection of a spouse is a process of men and women exchanging their resources to maximize respective interests [52]. Therefore, when an individual’s economic potential is low, he or she may rely on other attributes, such as attractive appearance, knowledge, or good character, to attract a mate with more money, so as to compensate for his or her own lack of economic resources. In addition, our study also found that individuals with higher resource-gaining capacity emphasized “good parent” traits more than those with lower resource-gaining capacity, and they did not value the resources of their partners. This finding suggested that “good parent” traits are “luxuries” in the short-term mating, and “luxuries” are traits that individuals would consider only in high-resource conditions [19]. Since the purpose of short-term mating is not to marry and raise offspring, “good parent” traits are not a “must” for individuals in the short-term mating.

Additionally, this study also found that men with lower resource-gaining potential showed an implicit preference for resource-related traits in implicit mate-selection decisions. The reason may be that women’s possession of resources is increasing with the development of the times, and they have greater economic potential. To reduce economic pressure, men with lower resource-gaining potential subconsciously pay more attention to the material conditions of their mates. Studies have found that, in some Western countries, women’s economic potential has begun to be an important consideration for men as women’s positions in the labor market continue to improve [53–55]. An increasing number of males choose their partners on the basis of education and socioeconomic status, and share the burden of family finances with their partners, especially when their employment situation is poor, as they cannot afford to rely on the traditional sex division of labor due to the increasing cost of living [56, 57].

### The effects of resource-gaining capacity and sex on mate preferences in long-term and short-term mating

Consistent with previous research, the results of explicit mate selection showed that women place more emphasis on “good provider” traits than men when choosing a long-term mate. In long-term mating, which involves parental investment and the provisioning of resources, individuals may seek a high-quality partner to assist with raising offspring. However, due to the increasing cost of marriage and raising children in modern society, the pressure associated with obtaining resources by oneself is greater, so it is more necessary to consider each other’s resource conditions. Studies have shown that the proportion of Chinese individuals who want their spouse to have a house and live in the city has increased over the years, that people weigh economic factors more than in the past, and that there is a salient tendency to seek practical benefits [58]. According to the structural powerlessness hypothesis in evolutionary psychology, men hold most of the social power and resources in most cultures, and women generally lack resources; thus, to ensure that they and their future generations obtain adequate and stable material resources and protection, women always have greater preferences for men who have access to more social resources and high-status positions [3, 13].

Besides, our study also found that women focus on “good parent” attributes more than men in the short-term mating. According to the parental investment theory, the reproductive benefits of short-term mating strategies for men are much greater than those for women, but the potential costs are smaller than those for women [59]. And the reproductive benefits of short-term mate selection for women are comparable to those of long-term mate selection strategies, but the potential costs are much greater than for men. Therefore, compared with men, women will be more cautious in choosing a short-term partner in order to pay as little cost as possible, and therefore pay more attention to “good parent” traits of their partner than men. Moreover, our study found that in regard to both short-term mating and long-term mating, men are more likely to value “good genes” traits than women. Good gene theory holds that good genes mean physical attractiveness, which means greater adaptability. It is thought that if an individual chooses a partner who is physically attractive, their offspring will also be more adaptable and competitive [60].

### Comparison of long-term and short-term mate selection

Our study found that individuals of both sexes preferred “good genes” more in short-term mating than in long-term mating, while they preferred “good parent” and “good provider” more in long-term mating than in short-term mating. This is consistent with previous researches [61–63]. According to stimulus-value-role (SVR) theory, different stages involve different exchange of resources. According to SVR theory, if the duration of mate selection behavior is short, then the resource exchange between the two partners only stays in the sensory stimulation stage, so they pay more attention to some immediate and enjoyable sexual luxury resources, such as looks and sexual attraction. However, if mate selection lasts for a long time and the mate selection stage develops into the value judgment stage or role expectation stage, then individuals pay more attention to the exchange of stable and unchangeable necessary resources, such as responsibility, income, social status and so on [64]. This is why individuals pay more attention to “good genes” in short-term mate selection than in long-term mate selection, while “good parent” and “good provider” are more important in long-term mate selection.

### Comparison of implicit and explicit mate selection

In this study, explicit mate selection cues were obtained by the participants clicking on trait words of different mating dimensions on the screen. The results showed that in both the short-term mating and long-term mating conditions, participants preferred “good parent” traits. “Good-parent” traits not only refer to the ability to raise offspring but also include other personality characteristics, such as gentleness, kindness and consideration. Especially in the college stage, the individual does not need to face many realistic problems, such as buying a house or marrying. When choosing a mate, individuals will give more consideration to personality factors and easily become partners based on factors such as having common hobbies and being in tune with each other [65]. Therefore, college students may attach more importance to “good parent” traits when choosing a partner.

Implicit mate selection was assessed by participants’ eye tracking data. Three eye tracking variables indicated that participants’ preferences for “good parent” traits were significantly lower than those for the other two dimensions in both short-term and long-term mate selection. To some extent, implicit (eye tracking) data indicated that the participants did not value “good parent” traits as much as they did in explicit (self-reported) mate selection; instead, they unconsciously preferred the “good provider” and “good genes” dimensions when choosing a mate. Because the explicit test was completed by the participants, with the participants selecting the trait words on their own, the participants may have been influenced by the social desirability bias to attempt to portray a positive image of themselves (i.e., that they assessed partners for internal attributes) [50]. In addition, individuals engage in conscious thinking and judgment in explicit mate selection. In this process, they are more vulnerable to the influence of traditional Chinese cultural concepts, pay more attention to conduct and spirit, and ignore some aspects such as material and appearance traits, which also generates differences between implicit and explicit test results. Therefore, there is still a strong sociocultural force in the Chinese cultural context, and the results of the explicit tests reflect the influence of this force. The results of the implicit eye tracking experiments, on the other hand, reflect the individual’s unconscious preference for mate selection and represent the most instinctive and real ideas in the depths of one’s heart. This implicit decision-making bias is driven entirely by evolutionary forces, with the ultimate goal being human reproduction and continuation [66]. Studies have shown that there is a low correlation between the results obtained by implicit and explicit testing [67], and the different results between the two tests reflect the different influences of the competition between the two forces of the social culture and biological evolution on mate selection.

### Limitations and perspectives

One limitation of this study is its small sample size (59 participants). Although post hoc power analysis revealed that the statistical power was 0.997, the sample size was small, which could limit the representativeness of the sample. In general, studies with a larger sample size are needed. Another limitation is that the participants were college students; these individuals are young and may not have a clear understanding of their specific requirements for long-term marriage partners.

Future studies should recruit a larger sample size and consider including young people of marriageable age as participants to explore the differences in their mate preferences under two different mating conditions, short term and long term. Additionally, the error caused by repeated measurements should be eliminated as much as possible. In addition, this study enhances understanding of young people’s mate preferences in the context of the Chinese culture, which extends sexual strategies theory. Moreover, these findings provide further insights into mate selection.

### Electronic supplementary material

Below is the link to the electronic supplementary material.

Supplementary Material 1: Research materials

## Data Availability

The datasets generated during and analyzed during the current study are available from the corresponding author on reasonable request.
