# Supplementary material for: The influence of resource-gaining capacity on mate preferences: an eye tracking study
Source: BMC Psychol. 2023 Dec 18;11:444. doi: 10.1186/s40359-023-01487-7 (PMC10726600; doi:10.1186/s40359-023-01487-7)
Supplement: Supplementary file 1 — Supplementary Material 1: Research materials [file 40359_2023_1487_MOESM1_ESM.docx]

Appendix 1:

**Basic Demographic Information Questionnaire**

1. Sex：

2. Major：

3. Grade：

4. Age： years months

5. How long have you been in a romantic relationship: days.

6. How many times have you been in romantic relationship in the past (including your current experience)： times

Appendix 2:

**Heterosexual-Homosexual Rating Scale**

Please make a truthful evaluation of your sexual orientation:

(no same-sex attraction at all) 1—2—3—4—5—6—7—8—9 (Extremely strong same-sex attraction)

Appendix 3:

**Resource-Gaining Capability Scale**

1. My future earning power will be:

(very poor) 1——2——3——4——5——6 (very good)

2. My future career potential will be:

(very low) 1——2——3——4——5——6 (very high)

Appendix 4:

**Words used in the eye movement experiment**

Male trait words:

“Good Genes” Group:

strong, healthy, handsome, brave, adventurous, innovative, humorous, nimble, physical, brave

“Good Fathers” Group:

tolerant, virtuous, filial, considerate, patient, dedicate, easy-going, honest, thrifty, open-minded

“Good Providers” Group:

powerful, extraction, wealthy, aspirant, diligent, ambitious, excellent, educated, potential, career

Female trait words:

“Good Genes” Group:

tender, healthy, beautiful, brave, adventurous, innovative, humorous, nimble, physical, bold

“Good Mothers” Group:

tolerant, virtuous, filial, considerate, patient, dedicate, easy-going, honest, thrifty, open-minded

“Good Providers” Group:

powerful, extraction, wealthy, aspirant, diligent, ambitious, excellent, educated, potential, career
